# Design, characterization and *in vivo* performance of synthetic 2 mm-diameter vessel grafts made of PVA-gelatin blends

**DOI:** 10.1038/s41598-018-25703-2

**Published:** 2018-05-09

**Authors:** M. Atlan, T. Simon-Yarza, J. M. Ino, V. Hunsinger, L. Corté, P. Ou, R. Aid-Launais, M. Chaouat, D. Letourneur

**Affiliations:** 1 INSERM U1148, Laboratory for Vascular Translational Science, X. Bichat Hospital, Paris Diderot University, Paris 13 University, 75018 Paris, France; 2 Faculty of Medicine, University Pierre et Marie Curie, Plastic Surgery Department, Hôpital Tenon, Paris, France; 3 MINES ParisTech, PSL Research University, MAT - Centre des Matériaux, CNRS UMR 7633, BP 87 91003, Evry, France; 4 ESPCI-Paris, PSL Research University, Matière Molle et Chimie, CNRS UMR 7167, Paris, 75005 France; 5 FRIM, INSERM UMS 034 Paris Diderot University, X. Bichat Hospital, 75018 Paris, France; 6 Plastic Surgery Department, Burn Unit, Paris Diderot University, Hôpital Saint Louis, Paris, France

## Abstract

Since the development of the first vascular grafts, fabrication of vessel replacements with diameters smaller than 6 mm remains a challenge. The present work aimed to develop PVA (poly (vinyl alcohol))-gelatin hybrids as tubes suitable for replacement of very small vessels and to evaluate their performance using a rat abdominal aorta interposition model. PVA-gelatin hybrid tubes with internal and external diameters of 1.4 mm and 1.8 mm, respectively, composed of 4 different gelatin ratios were prepared using a one-step strategy with both chemical and physical crosslinking. By 3D Time of Flight MRI, Doppler-Ultrasound, Computed Tomography angiography and histology, we demonstrated good patency rates with the 1% gelatin composition until the end of the study at 3 months (50% compared to 0% of PVA control grafts). A reduction of the patency rate during the time of implantation suggested some loss of properties of the hybrid material *in vivo*, further confirmed by mechanical evaluation until one year. In particular, stiffening and reduction of compliance of the PVA-gelatin grafts was demonstrated, which might explain the observed long-term changes in patency rate. These encouraging results confirm the potential of PVA-gelatin hybrids as ready-to-use vascular grafts for very small vessel replacement.

## Introduction

Since the construction of the first tissue engineered blood vessels^1^, using a Dacron mesh as support, there are still only two types of synthetic materials used in the clinic for vessel replacement, namely polyethylene terephthalate (Dacron®) and polytetrafluoroethylene (PTFE)^2^. However, the use of these materials as first choice strategy is only established for middle to large vessels with diameters greater than 6 mm. For smaller vessels the gold standard is the autograft using patient saphenous vein (SV) or internal thoracic artery (ITA). Nevertheless, issues related to the use of autologous grafts are numerous: autologous vessels are not always available, their quality is often compromised and the extraction causes donor site morbidity. Moreover, autografts present important failure rates, as in the case of SV grafting with coronary artery bypass grafting and femoropopliteal bypass grafts that performed 50% failure rate at 10 years^3,4^. Despite promising tissue-engineering studies and intensive industrial development on the last decade on arterio-venous shunts for haemodialysis using autologous or allogenic cells, these products did not reach the market^5–8^. It is thus appropriate to develop ready-to-use materials that can be used as synthetic vascular grafts with diameters less than 6 mm.

Numerous materials are being explored, to be used as vascular grafts for small vessel replacement, such as polycaprolactone^9^, polylactide^10^, bi-hybrid polyurethane/poly (ethylene terephthalate)^11^, bacterial nanocellulose^12^ and decellularized aorta from animals^13^. In the last years, promising results by our group^14^ and others^15,16^ have already been obtained for the development of vascular grafts based on poly (vinyl alcohol) (PVA). PVA has demonstrated to be an attractive synthetic polymer due to the combination of important properties such as availability, biocompatibility and low cost. It has been proposed for numerous tissue engineering applications, namely replacement of the cornea^17^, vitreous^18^, cartilage^19^, ligament^20^, skin^21^ and cardiac valve^22^. In order to fulfil the requirements to be used as vascular graft, a previous work explored the combination of films of PVA with gelatin to prepare 2D hybrid materials^14^. Very satisfactory results were obtained in terms of mechanical properties, haemocompatibility and endothelial cell adhesion. The present work aims to further develop PVA-gelatin hybrids as tubes suitable for small vessel replacement (<2 mm) and to evaluate their performance as vascular grafts in an animal model. Using an innovative strategy in one step with both chemical (sodium trimetaphosphate, STMP) and physical (freezing) crosslinking, PVA-gelatin hybrid blend tubes with different gelatin ratios have been prepared and characterized. A first short *in vivo* study was performed to evaluate graft patency and select the best composition (1% gelatin) for a longer 3-month *in vivo* evaluation. Those *in vivo* performances at 3 months were assessed using several imaging techniques and histological evaluation. They were completed by measurement of released gelatin and mechanical characterization under *in vivo*-simulated conditions to investigate the effect of material aging.

## Results

### Grafts preparation and characterization

**Figure 1:**
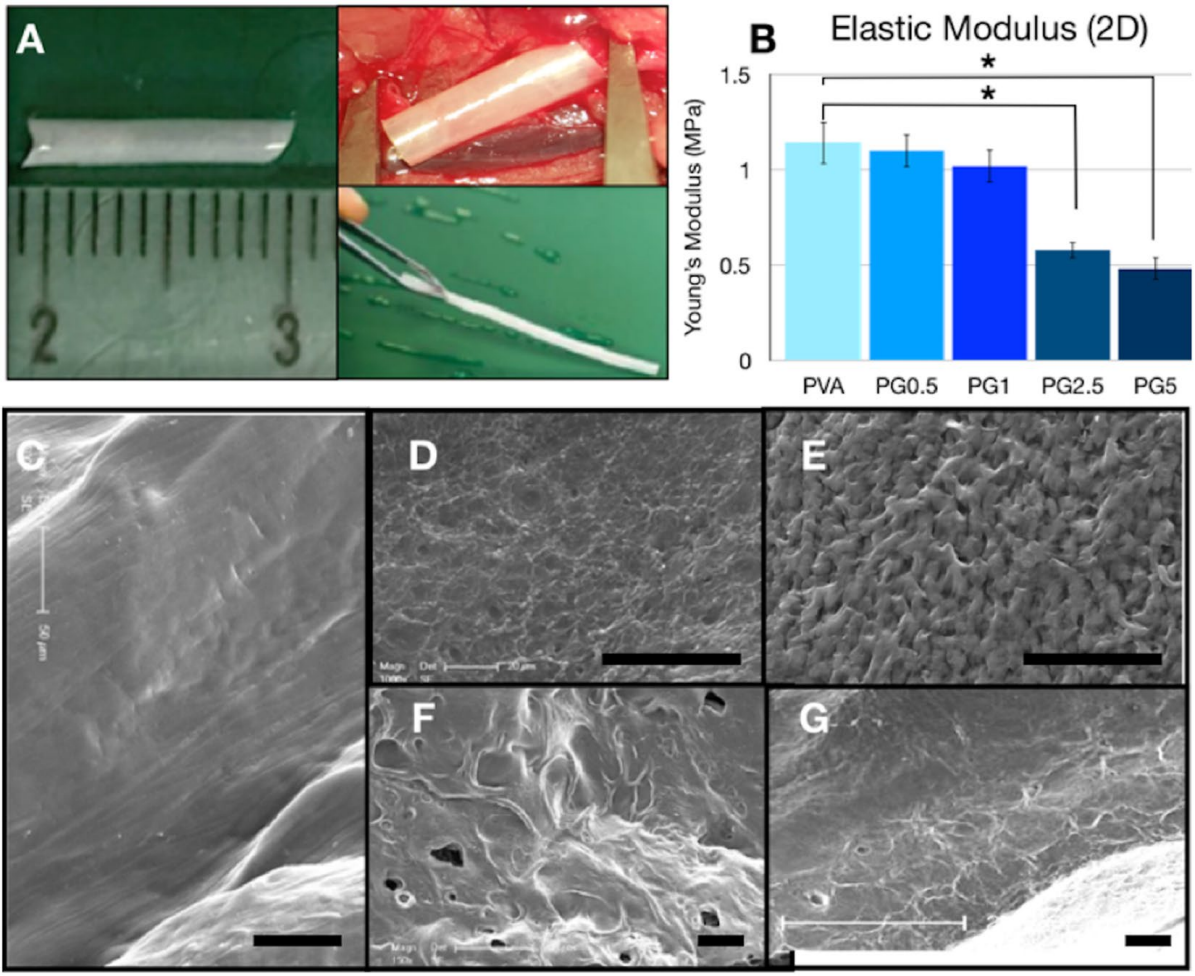
(**A**) PVA tube of 1 cm and handling of the grafts for the vascular replacement at the level of the abdominal aorta was easy and without collapse of the lumen. (**B**) Elastic modulus of PVA, PG0.5, PG1, PG2.5 and PG5 dry membranes measured at room temperature after less than 48 h after their manufacturing. (**C**–**G**) Scanning electron microscopy images of vascular grafts. Luminal surface of PVA (**C**) presented smooth surface, while addition of gelatin was associated to the formation of microscale irregularities in the luminal side (**D**–**G**). In the case of PG0.5 (**D**) and PG1 (**E**) quite homogeneous pores of 15–30 μm were observed, whereas higher gelatin content led to ridges larger than 100 μm (**F**: PG2.5; **G**: PG5). Scale bar: 50 μm. Graph represents average ± s.e.m.; *p < 0.05.

All the PVA and PVA-gelatin solutions were transparent, as well as the materials obtained after chemical cross-linking. Physical crosslinking by freeze-drying caused macroscopically visible changes and the 2D films and tubes became white and opaque, even after rehydration in PBS (Fig. 1A). There were no macroscopically detectable differences within the samples with different compositions. None of the tubes collapsed during the preparation or under storage conditions. The mean size of the outer diameter and inner diameter was very homogenous: 1.8 ± 0.4 mm and 1.4 ± 0.2 mm respectively (n = 8). Manufacturing process was highly reproducible and led to stable results for the dimensions and the macroscopic appearance.

**Figure 2:**
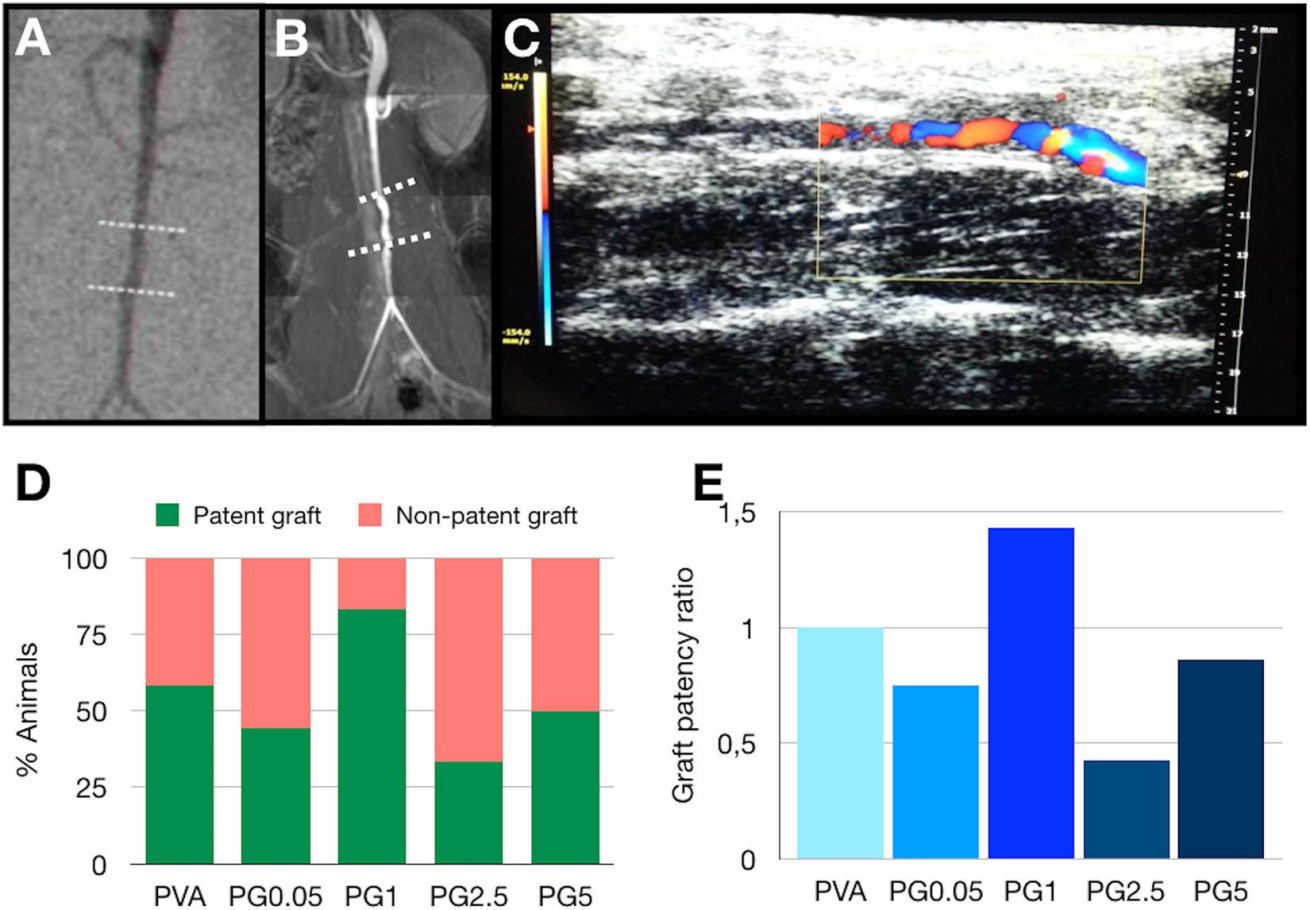
Graft patency evaluation. Three different techniques were used for patency evaluation: CT angiogram (**A**), TOF-MRA (**B**) and Doppler-US imaging (**C**). Graft patency after 7 days expressed as % of animals with patent or non-patent grafts (**D**) and as graft patency ratio compared to PVA control group (**E**). In A and B discontinuous white line indicates the presence of the graft. In C blood velocity within the implant is represented by a colour scale.

SEM images revealed that the addition of gelatin caused changes on the luminal surface of the grafts (Fig. 2C–G). PVA surface was smooth (Fig. 1C) whereas important roughness was observed for all the PVA-gelatin hybrids (Fig. 1D–G) with a different surface structuration clearly dependent on the gelatine content. Indeed, lower gelatin content was associated to small micropores with an average size of 15–30 μm, as observed for PG0.5 and PG1 (Fig. 1D,E, respectively), and samples with the highest gelatin contents presented larger and more heterogeneous microdomains, with elevated ridges of 100 μm and pores within 100 and 200 μm (see Fig. 1F for PG2.5 and Fig. 1G for PG5).

Elastic Moduli of all the materials as 2D membranes were calculated in their dried state at room temperature (Fig. 2B). Addition of gelatin was associated to a decrease of the elastic modulus that was concentration-dependent. Indeed, the slight non-significant decrease observed for PG0.5 and PG1 became much greater in the case of PG2.5 and PG5 whose elastic moduli were significantly lower compared to the PVA control group.

Suture retention, compliance and burst pressure were studied after graft preparations (Table 1). Suture retention (>50 gmf) was lower than that of human vessels but compatible for human application^23^. Burst pressure values for PVA and PG1 grafts were more than twice higher than the human healthy systolic arterial pressure. Graft compliance was very similar to those of native vessels (Table 1).

**Table 1:** Burst pressure, suture retention and compliance of PVA and PG1 as prepared, PG1 after 2 months incubation in PBS at 37 °C.

	Burst pressure (mm Hg)	Suture retention (gmf)	Compliance (%)	Elastic modulus (MPa)
PVA	703 ± 17 (n = 6)	93 ± 4 (n = 4)	3.0 ± 0.4 (n = 5)	1.38 ± 0.10 (n = 5)
PG1	614 ± 21 (n = 6)	83 ± 6 (n = 4)	3.9 ± 0.4 (n = 5)	0.783 ± 0.07 (n = 5)
PG1 (8 weeks PBS 37 °C)	802 ± 21 (n = 6)	104 ± 9 (n = 4)	3.3 ± 1.4 (n = 5)	1.09 ± 0.06 (n = 5)
Rat abdominal aorta	>3,000^24^	107 ± 11^24^	6.4 ± 0.4^24^	—
Human internal thoracic artery	2,031–4,225^5^ (n = 13)	200 ± 119 (n = 9)^5^	4.5–6.2^5^	0.27 ± 0.05
Human saphenous vein	1,680–2,273^5^	196 ± 2 (n = 7)^5^	4.4 ± 0.8^28^	0.068 ± 0.15

Parameter values of some rat and human arteries and veins commonly used as grafts are included as reference values (when known the number of analysed samples is indicated). Data for rat and human blood vessels were reported elsewhere^5,24,28^.

### *In vivo* tests

All animals included in the short and long-term study were sacrificed at the established time points. Until sacrifice all animals in the study remained in good health and presented no sign of local or systemic infection and no visual ischemia of legs except for one abdominal abscess and one abdominal wound dehiscence not related to the prosthesis.

### Short-term study

All grafts were successfully implanted without any problems concerning artery size mismatch, burst or dilation of the implants, or suture complications.

A 7-day *in vivo* assay was performed to assess PG5, PG2.5, PG1 and PG0.5 graft patency of grafts with diameter <2 mm and without any anticoagulation treatment. It showed an improvement compared to the PVA control group only for the PG1 implant. Evaluation by Doppler-US (Supplementary Video 2), CT angiography and TOF-MRA (Supplementary Video 3) (Fig. 2A–C) showed 58% patency rate of the PVA control group (n = 12). PG0.5 (n = 9), PG2.5 (n = 9) and PG5 (n = 12) revealed lower values of graft patency, equivalent to 44%, 33% and 50%, respectively (Fig. 2D). Only the grafts prepared with 1% gelatin (n = 12) resulted in an improvement of the patency that was 83%, which corresponds to a 1.43 improvement rate compared to the PVA control group (Fig. 2E). Based on these results, PG1 was chosen for the long-term *in vivo* studies.

### Long-term study

After 4, 8 and 12 weeks, four animals in PVA and PG1 groups were randomly selected for graft evaluation by imaging. Immediately after, those animals were sacrificed to compare the results of *in vivo* imaging to histological observations.

**Figure 3:**
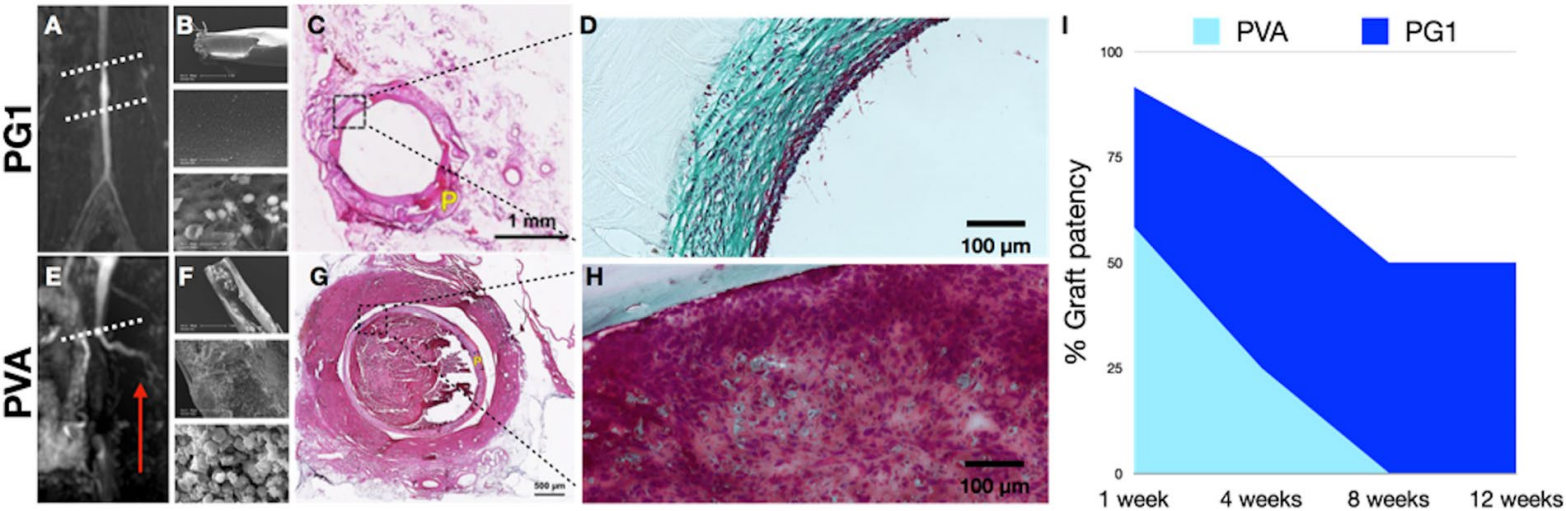
Long-term *in vivo* study. TOF-MRA allowed the identification of patent and thrombosed grafts (white discontinuous line in **A** and **E** respectively). TOF-MRA evidenced the development of collateral vasculature in animals with thrombosed grafts (red arrow in **E**) (image corresponds to 2 months time point). After graft explantation, SEM analysis of patent grafts (**B**) and non-patent grafts (**F**) confirmed the presence of thrombus in the lumen of the tubes in the case of non-patent grafts. Hematoxylin-eosin staining of the harvested implants (**C** and **G**) clearly evidenced the thrombus in the tube lumen (**G**), as well as new tissue formation within patent tubes (**C**). Masson’s trichrome staining confirmed in green the presence of an organised extracellular matrix in the case of patent grafts (**D**) compared to the non-patent grafts (**H**), where collagen deposits appeared in the thrombus. Imaging and histology throughout 12 weeks showed a clear improvement of graft patency in PG1 group compared to the control PVA group (**I**). P in yellow stands for vascular Prosthesis.

Graft imaging revealed that after one month only 25% of animals in the PVA control group presented graft patency whereas in the PG1 group this value was 75%, (3-fold improvement compared to the PVA control group). The higher performance of PG1 grafts was confirmed after 8 and 12 weeks, when 100% of the animals in the PVA control group had occluded grafts, while in the PG1 group 50% of animals still presented graft patency (Fig. 3H).

**Figure 4:**
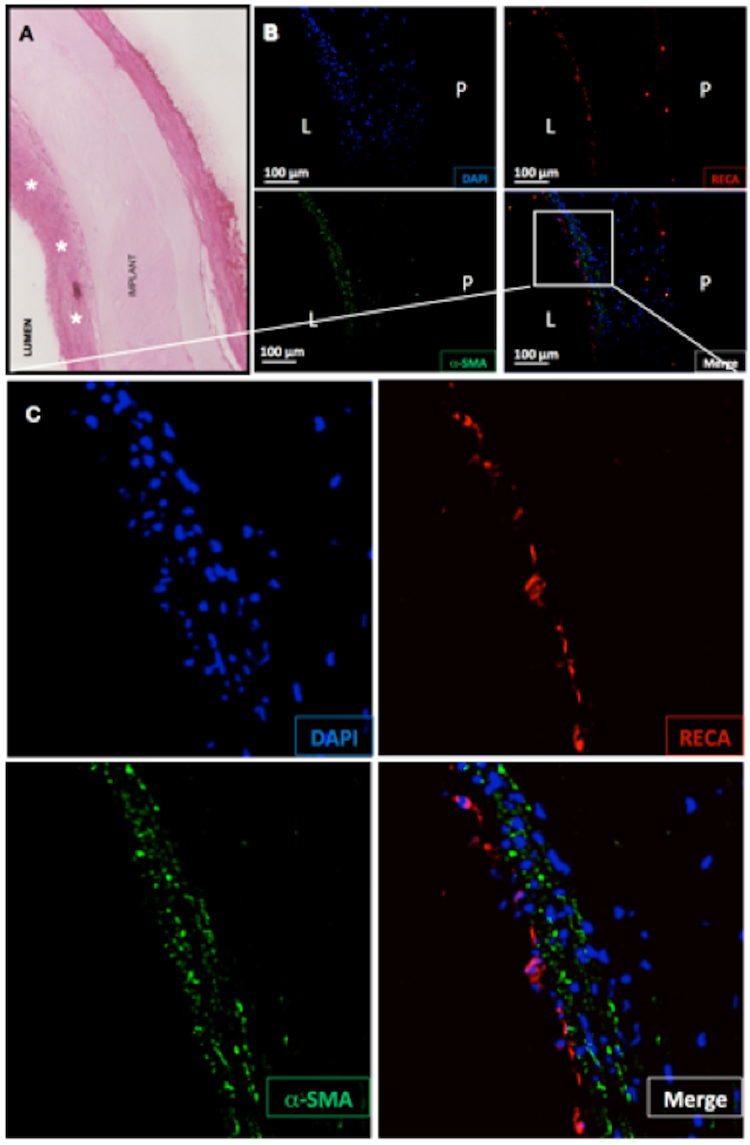
PG1 patent tube 12 weeks after implantation. Hematoxylin-eosin (**A**) demonstrated the presence of multilayered cellular organization, with a confluent newly formed tissue (white stars). Any cell infiltration was observed within the graft. Immunofluorescence images (**B** and **C**) confirmed the presence of endothelial cells in red (RECA) and smooth muscle cells in green (alpha-SMA) within the endothelium. (Nucleus stained in blue).

All the patent grafts presented a good integration with the adjacent arteries. Grafts were found encapsulated in an inert 200–300 µm capsule (Supplementary Figure 1A). They did not present signs of degradation and maintained their tubular shape throughout the whole study (12 weeks). Aneurysm, dilation or any other major deformation was not observed in any of the implants (Supplementary Figure 1B). Any cell infiltration within the graft was observed, consistent with the lack of porosity of the PVA tubes (Fig. 4A).

TOF-MRA led to high definition images that allowed observing the development of collateral vascular network already after 2 months in those animals presenting thrombosis (Red arrow in Fig. 3E). Histological evaluation of the grafts always corresponded to the imaging-based diagnosis made by the 3 observers (Fig. 3A–G). Hematoxylin-eosin staining showed the development of a confluent endothelium in 70% of the PG1 patent grafts (Figs 3C and 4A). The tissues analysed by immunofluorescence demonstrated the formation of recognizable vascular tissue with a wall of smooth muscle cells and a luminal endothelial cell layer (Fig. 4B).

**Figure 5:**
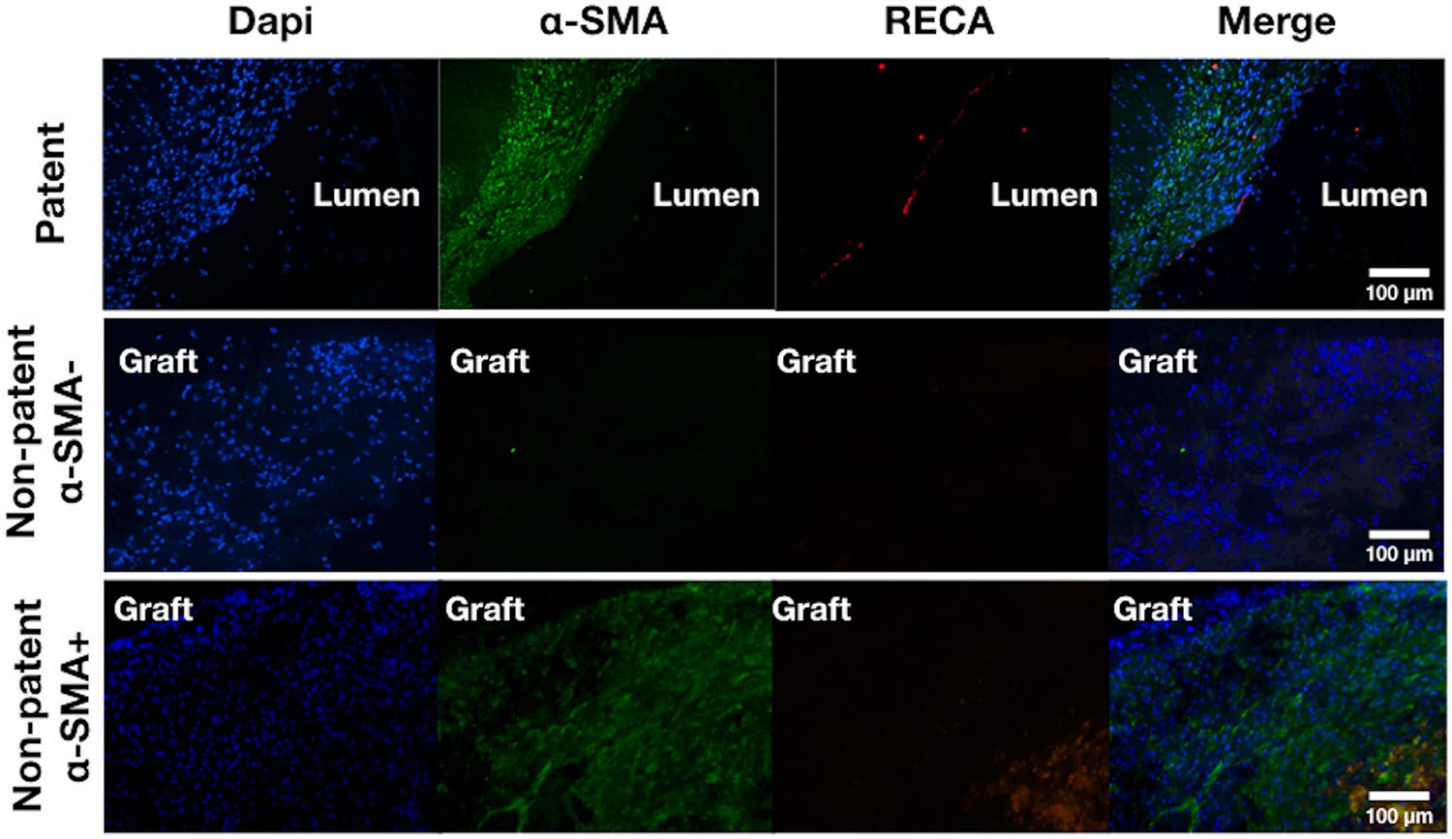
Representative pictures of tissues following graft implantation. Patent grafts presented new tissue formed within the lumen characterized by α-SMA+ and RECA+ cells. In the case of non-patent grafts, α-SMA+ cells were found only in some cases, and cells were always RECA− (note that the red staining in the case of Non-patent SMA+ corresponds to autofluorescence of red blood cells in the thrombus.

Further histological analysis allowed us to identify three different types of tissue evolutions after graft implantation (Fig. 5). In the case of PG1 patent grafts after three months, immunostaining confirmed the presence of a newly formed tissue with well-organized collagen fibers (Fig. 3D), slight hyperplasia, smooth muscle cells and endothelial cells. Besides, within the animals with non-patent grafts, histology revealed two different histological patterns, one characterized by the absence of smooth muscle cells and the other one presenting sparse smooth muscle cells next to the implant. In both cases, immunostaining was negative for endothelial cells. This suggests that after implantation, thrombosis occurred in some of the grafts. The other prostheses favoured the development of new tissue formation within the lumen of the graft. When this tissue was endothelialised, the graft remained patent, whereas in late thrombosed grafts endothelial cells were absent. PG1 grafts favoured the formation of SMA+ tissue in the grafts compared to PVA, as well as endothelialisation that was observed only in PG1 grafts.

Alizarin staining revealed progressive calcification after three months and some deposits of calcium phosphate could be detected. Other studies performed in the laboratory have demonstrated that calcification occurs after one year in a greater extent, even in patent grafts (results not shown), indicating that calcification is not always associated to graft failure. Finally, elastin fibers were not detected after orcein staining in all our samples.

### Characterization of PVA-Gelatin 1% grafts

*In vivo* results demonstrated a clear benefit of the addition of 1% gelatin to the PVA prostheses. However, the graft patency observed after 1 week (83%) and 4 weeks (75%) was reduced after 8 and 12 weeks (50%) (Fig. 3H). There was therefore a negative tendency suggesting that the PG1 implants lose some of their properties after being implanted *in vivo*. To better understand this phenomenon, PG1 was further characterized under *in vivo* simulated conditions up to 12 weeks, to mimic the conditions of the long-term study.

**Figure 6:**
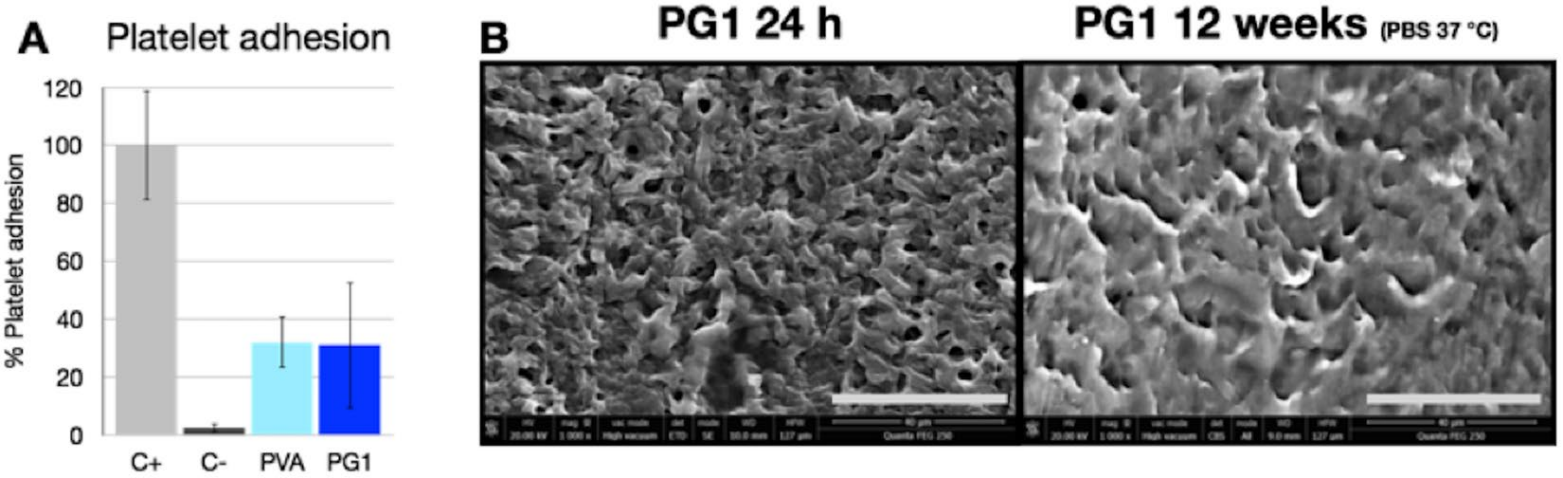
(**A**) Platelet adhesion on the PVA and PG1 surface. Collagen positive control group (C+) and BSA negative control group (C−) were used. Graph represents average ± s.e.m. (**B**) Luminal surface of PG1 tubes as prepared (24 h) and 2 months after incubation in PBS at 37 °C. Scale bar: 40 μm.

Firstly, the interactions of PG1 and PVA with blood platelets were compared (Fig. 6A). In both cases, platelet adhesion was found to be greater than in the case of the BSA negative control group, but it remained significantly lower than the collagen positive control group. Nevertheless, there were no differences between the two materials, indicating that the thrombogenicity seems not responsible for the differences of *in vivo* performances between PVA and PG1.

The simple method to incorporate the gelatin to the PVA material consisted in physical addition, which means that the gelatin was not covalently linked to the PVA. Thus, gelatin release under simulated physiological conditions was studied, to confirm that the gelatin remained present in the implant for a time sufficient to promote lumen endothelialisation. Slow release of the gelatin was confirmed with only 4.6 ± 0.8% of the total gelatin content released after one week. After 12 weeks more than 50% of the gelatin still remained in the prostheses. This partial release can explain the differences on the PG1 luminal surface observed by SEM after incubation of PG1 for 12 weeks in PBS at 37 °C, characterized by slight changes of the pore size and of the roughness of the material (Fig. 6B).

### Mechanical properties under simulated *in vivo* conditions

Suture retention, compliance and burst pressure data of PVA and PG1 as prepared and after 2 months incubation under physiological simulated conditions are compiled in Table 1. PVA and PG1 presented adequate mechanical properties to be used as vascular grafts. Suture retention remained in all cases higher than 50 gmf, value established as satisfactory for surgery^23^. PG1 as prepared showed slightly lower suture retention (83 ± 6 gmf) compared to PVA (93 ± 4 gmf), but the difference was not significant. After 2 months incubation in PBS at 37 °C, suture retention of PG1 was significantly increased up to 104 ± 9 gmf, higher than that of PVA. Graft compliance was in all cases very similar to those of ITA and SV. In the case of grafts evaluated after preparation, compliance was higher for PG1 (3.9 ± 0.4%) compared to PVA (3.0 ± 0.4%), but this difference was decreased after 2 months, with PG1 compliance of 3.3 ± 1.4%. Similarly, PG1 initial burst pressure was inferior to PVA value (614 ± 21 mm Hg and 703 ± 17 respectively), but after 2 months incubation, PG1 burst pressure significantly increased to 802 ± 21 mm Hg. In all cases, this represented more than twice the human healthy systolic arterial pressures.

**Figure 7:**
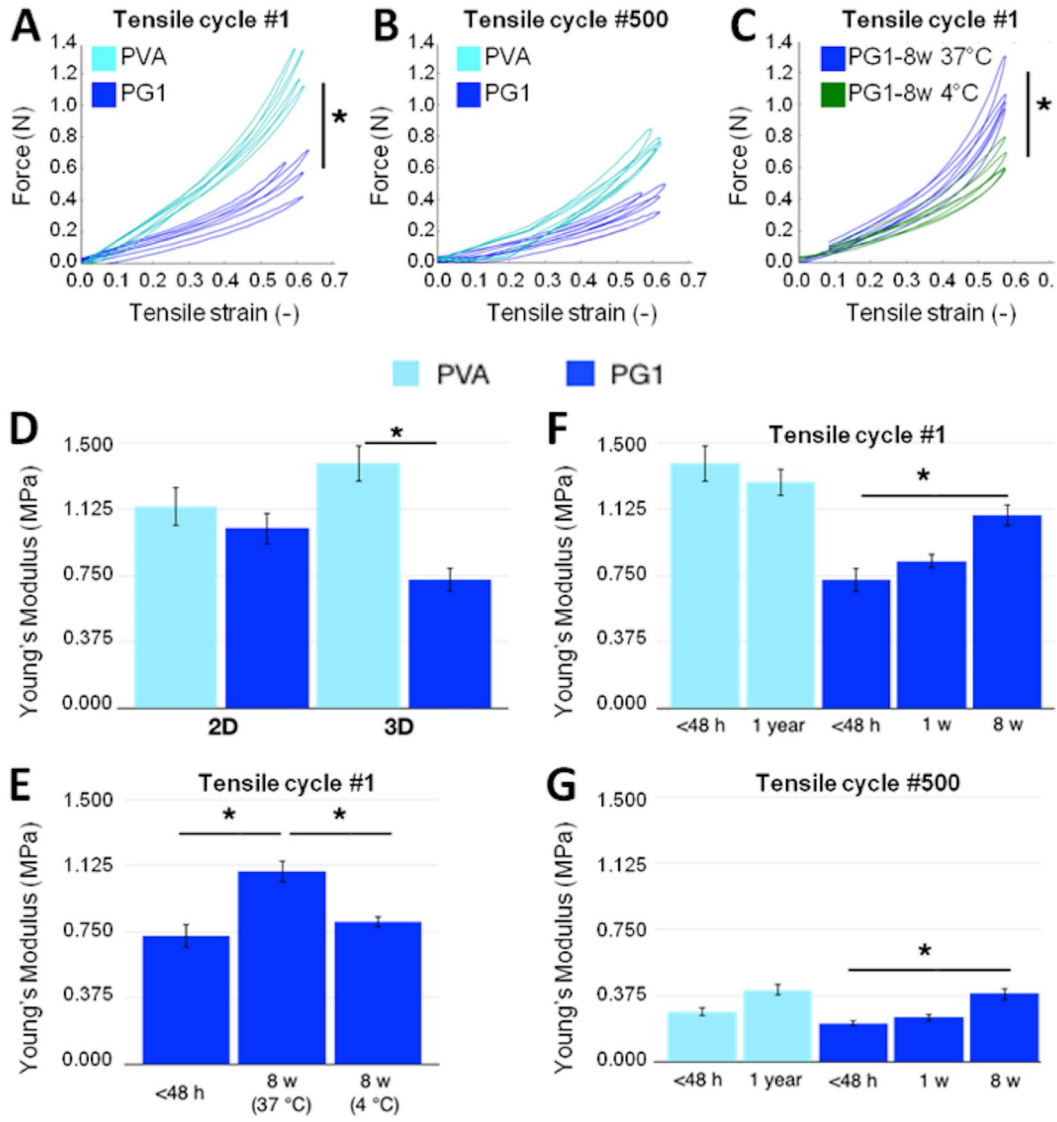
Evolution of the mechanical properties of PVA and PG1grafts under physiological simulated conditions. (**A**,**B**) Force-strain curves of PVA and PG1 grafts measured less than 48 hours after fabrication for the 1^st^ tensile cycle (**A**) and the 500^th^ tensile cycle (**B**). (**C**) Force-strain curves of PG1 grafts measured after 8 weeks in PBS at 37 °C or 4 °C (1^st^ tensile cycle). (**D**) Comparison of the Young’s elastic moduli of PVA and PG1 prepared as membranes (2D) or as tubular grafts (3D) in PBS 37 °C less than 48 hours after fabrication. (**E**) Elastic Modulus of PG1 3D grafts measured less than 48 hours after fabrication, and after 8 weeks in PBS at 37 °C or 4 °C. (**F**,**G**) Young’s modulus of PVA and PG1 3D grafts measured less than 48 hours after fabrication and after different storage times in PBS at 37 °C for the 1^st^ (**F**) and the 500^th^ (**G**) tensile cycle. Graphs represent average± s.e.m.; *p < 0.05.

Ageing of the material, *i.e*. the evolution of the implants mechanical properties under simulated physiological conditions, was assessed using uniaxial tensile testing in physiological serum at 37 °C (Supplementary Video 4). The tensile force-strain curves for the 1^st^ and 500^th^ cycles are shown in Fig. 7A,B, respectively, for both PVA and PG1 tubes. All grafts exhibited a non-linear elastic response and negligible permanent elongation even after 500 cycles. PVA tubes were significantly stiffer than PG1 tubes. This difference was still observed after 500 cycles even though both PVA and PG1 tubes get substantially softer.

Besides, the values of Young’s modulus for tubes (3D) were compared to those for films (2D) in Fig. 7D. As previously mentioned, the elastic modulus in 2D films was similar for both PVA (1.14 ± 0.11 MPa) and PG1 (1.02 ± 0.09 MPa). On the contrary, for tubes under *in vivo*-like conditions (physiological serum at 37 °C), PVA tubes had a higher modulus than PG1 tubes and the elastic modulus was higher for PVA tubes (1.39 ± 0.10 MPa) and lower for PG1 tubes (0.73 ± 0.07 MPa) as compared to 2D values.

To perform a deeper evaluation of the effect of aging on the mechanical properties of the grafts, tubes of PVA and PG1 were kept at 37 °C in PBS and were tested mechanically with the same protocol after different storage times. The corresponding elastic moduli are shown in Fig. 7F and G for the 1^st^ and the 500^th^ cycles, respectively. PVA tubes kept a stable elastic modulus over time, with little differences even after 1 year storage. On the contrary, PG1 tubes were found to become significantly stiffer with time, no matter the number of cycles, with a modulus increasing from 0.73 ± 0.07 MPa to 1.09 ± 0.06 MPa after 2 months of incubation. Interestingly, the values of Young’s moduli for PG1 tubes were found to have the same order of magnitude as human internal thoracic artery values.

The effect of aging on the mechanical properties was further characterized by comparing PG1 tubes after 2 month storage at 37 °C and at 4 °C, well below the melting point of gelatin. The corresponding tensile force-strain curves are shown in Fig. 7C for the 1^st^ tensile cycle. The tensile response of the grafts after storage at 4 °C was found to be significantly softer than that of the grafts stored at 37 °C during the same time. In particular, the values of the Young’s modulus for 4 °C storage remains very close to the values measured right after fabrication, as shown in Fig. 7E. The stability of the mechanical behaviour at 4 °C suggests that the graft microstructure and composition are preserved while they are progressively altered under *in vivo*-like conditions.

## Discussion

In the reported study, a <2 mm off-the-shelf prosthesis with *in situ* endothelialisation was preferred to vascular pre-seeding to alleviate the difficulties associated to *in vitro* cell culture, variability associated to autologous strategy or immunological response with allogenic cells, requirement of specialized equipment and personals^2^.

The preparation method described here allowed the fabrication of 1.4 mm internal diameter tubes and 400 μm wall thickness (rat aorta thickness), in an easy, reproducible and inexpensive fashion. Combination of PVA and gelatin to form hybrid 3D prostheses aimed at taking advantage of the relevant mechanical and chemical properties of both materials. Considering that the three principal causes of graft failure, namely thrombosis, intimal hyperplasia and atherosclerosis, are intimately related to incomplete lumen endothelialisation and compliance mismatch between the prosthesis and the native vessel, gelatin was chosen to assure endothelial cell adhesion on the lumen of PVA tubes that possess appropriate mechanical properties for vessel replacement^24^. Keeping in mind that the preparation method consisted of only one freeze-thaw step, one could expect a low elastic modulus not suitable for implantation. Other authors have reported PVA cryogel preparation with three or more freeze-thaw cycles^25^. The high elastic modulus obtained here is the result of two simultaneous ways of crosslinking, physical and chemical, mediated by freeze-thaw cycles and the crosslinker STMP, respectively, previously described^14,24^, together with the use of a high molecular weight PVA (84–124 kDa). Addition of gelatin, which is softer than PVA, decreased the Young’s modulus of implants (Fig. 6A), but the mechanical properties appeared to be dominated by PVA, in accordance to previous observations by Liu *et al*.^26,27^. Suture retention of PVA and PG1 tubes was found to be lower than that of human vessels but compatible for human application, remaining in all cases over 50 gmf, the minimal value considered satisfactory to avoid any surgical or post-implantation anastomotic complications^23^. PG1 burst pressure was inferior to PVA value (614 ± 21 mm Hg and 703 ± 17, respectively) but both presented values more than twice the human healthy systolic arterial pressure. Graft compliance for both PG1 and PVA grafts (PG1 3.9 ± 0.4% and PVA 3.0 ± 0.4%) was very close to that of the human ITA (4.5–6.2%) and human SV (3.6–5.2%)^5,24,28^ (Table 1). In particular, compliance of PG1 (3.9 ± 0.4%) was well above those of clinical synthetic grafts PTFE (1.6 ± 0.2%) and PET (1.9 ± 0.3%), as reported in the literature^28,29^. Compliance mismatch between synthetic grafts and native arteries has been demonstrated to be a determining factor for long-term graft patency. Indeed, Salacinski *et al*. performed linear regression analysis of compliance values and patency rates of various biological and prosthetic grafts, demonstrating a highly significant correlation between compliance mismatch and graft patency decrease^28^. Higher patency rates were associated to compliance values near to 4%, as those observed for PG1 prostheses in this study, whereas PTFE and PET compliance values near 2% were associated to the lower patency rates.

A previous study already demonstrated that PVA-gelatin films promoted *in vitro* endothelial cell adhesion compared to PVA films^14^. This phenomenon was associated to both chemical and topographic changes. Indeed, gelatin is well known to possess in its structure adhesive cell motifs such as Arg-Gly-Asp, but it also causes concentration-dependent modifications on the surface topographies (Fig. 1C–G) that are known to modulate cell adhesion^30^.

Addition of 1% gelatin to PVA on model 2D surfaces increased cell adhesion, compared to 5% and 10% gelatin ratios^14^. This was attributed to heterogeneous distribution of gelatin that accumulated on the edges of the micro-domains observed with electron microscopy (Fig. 1F,G). These previous results are consistent with *in vivo* observations in the present work and could explain the better performance of PG1 vascular grafts after one week, due to faster *in situ* endothelialisation of the graft and prevention of graft failure. Moreover, presence of 1% gelatin in the graft allowed better patency results after one week (83%) than with PVA-heparin hybrid implants (not included in this work) that caused only 62% patency rate. This suggested that accelerated *in situ* endothelialisation of PVA implants by gelatin was an efficient strategy to avoid thrombosis. Moreover, since platelet adhesion of PG1 did not differ from PVA (Fig. 5A), it is possible to conclude that platelet did not trigger the PG1 better outcomes.

Long-term *in vivo* studies confirmed the excellent capabilities of <2 mm PG1 vascular grafts without anticoagulant treatments of animals, with patency rates of 75% after 4 weeks and 50% after 8 and 12 weeks. This work demonstrates an improvement compared to a previous study by some of us^24^, were PVA tubular grafts fabricated by gluing or stitching a PVA film were followed *in vivo* during 7 days. Indeed, to the best of our knowledge, this is the longest and best patency rate reported in the literature for PVA grafts.

In this work graft patency was evaluated by 3 different imaging techniques. TOF-MRA was successfully employed and provided reliable information on patency without the use of any contrast agent. TOF-MRA was a fast technique that did not present evaluation limits due to low vessel diameter and that was not operator-dependent as in the case of Doppler-US. Histology confirmed imaging-based diagnosis in 100% cases. Moreover, it demonstrated a high rate of *in situ* endothelialisation with 70% of PG1 patent grafts in the study presenting a confluent endothelium as well as the formation of recognizable vascular tissue with a wall of smooth muscle cells and a luminal endothelial cells layer (Fig. 4B).

The increase of thrombosis at late time points (50% at 2 and 3 months) suggested a decrease of PG1 performance as vascular graft over time. The phenomenon of gelatin release could not explain graft failure since it was confirmed in other studies in the laboratory (results not shown) that new tissue formation occurs during the first 7 days after implantation, and at that time less than 5% gelatin released occurred *in vitro*. After 8 weeks more than 50% of gelatin was still found within the implant. This release was accompanied with slight changes of the topography (Fig. 5B) that could not justify either graft failure.

After implantation, grafts evolve and suffer remodelling or aging that can drastically modify chemical and mechanical properties. Even if harvested implants were found intact and without any signs of degradation or morphological changes, mechanical evaluation after storage under physiological conditions (PBS, pH 7.4, 37 °C) demonstrated important changes on the mechanical properties. Results showed that PG1 after manufacturing was more flexible and compliant than PVA, but the aging process transformed it into a stiffer material after 2 months at 37 °C, becoming a graft with similar mechanical properties to original PVA. These results were confirmed at cycle 1 and cycle 500 (Fig. 6F,G). Several tensile cycles were applied to simulate mechanical fatigue and the number of cycles resulted in important changes in the elastic moduli both for PVA and PG1, whose elastic moduli were significantly reduced with the number of cycles (Fig. 6F,G). In this aging process, temperature played a central role as demonstrated by the evaluation of PG1 grafts stored for 2 months in PBS at 37 °C or 4 °C (Fig. 6E). Elastic modulus of PG1 after 2 months of storage at 4 °C was similar to the value of PG1 after preparation. Together with measurements of gelatin release, these mechanical results suggest that the changes in mechanical properties *in vivo* are related to some modifications of the gelatin phase which melts at 37 °C and leaks out of the grafts. All together, results indicate that the initial good mechanical properties of PG1 to be used as a vascular graft are lost under *in vivo* conditions. This lost of mechanical properties is consistent with the decrease of graft patency for PG1 implants over time. This is also supported by histological results that revealed that in some cases, prior to thrombosis, new tissue formation within the graft occurs. Indeed, after implantation the graft presents the appropriate mechanical properties to assure tissue formation. In a latter time, thrombosis occurs, probably due to the lost of mechanical properties.

In conclusion, tubular grafts of 1.4 mm in diameter and thickness of the rat aorta made of PVA with 1% gelatin were prepared through a double crosslinking process that gave rise to mechanical properties very close to those of human autologous gold standards. Grafts were evaluated *in vivo* and allowed *in situ* endothelialisation, as well as the formation of recognizable vascular tissue with a wall of smooth muscle cells and a luminal endothelial cells layer, confirming that gelatin at the appropriate dose is a major factor to initiate *in situ* cell adhesion. Endothelialisation was accompanied by high patency rates after 1, 4, 8 and 12 weeks, 100%, 75%, 50% and 50% respectively. After 8 and 12 weeks graft failure augmented, possibly due to changes in the mechanical properties, as suggested by the evolution of the mechanical properties under *in vivo* simulated conditions. Graft evolution is the result of multiple factors and varies from one individual to another, being not possible to identify an exclusive cause of graft failure. Results in this study highlight the importance of PVA-gelatin graft aging that must be taken into account for future works in order to reach very long-term graft patency.

## Materials and Methods

### PVA and PVA-gelatin hybrid graft preparation

Grafts were prepared using a chemical crosslinking process adapted from a previous study^14^. Briefly, PVA (Mw 84–124 kDa, saponification degree >99.9%) and gelatin type B from bovine skin (Sigma, G9391, 225 Bloom number) were dissolved in water under magnetic stirring at 90 °C for 3 h. Different gelatin concentrations were set at 0%, 0.5%, 1%, 2.5% and 5% (w/w) and labelled PVA, PG0.5, PG1, PG2.5 and PG5 respectively. Once the solution was cooled down to room temperature, nine grams of the solution were progressively mixed with 750 μL of 15% (w/v) crosslinker STMP and 300 μL of 30% (w/v) sodium hydroxide under magnetic stirring. To avoid air bubbles, the blend was centrifuged (4,300 rpm for 10 min). The PVA and PVA-gelatin solutions were cast in a tubular mold centered by a rod and covered with a silicon cap, and stored at −80 °C for 15 min to allow gelation. Then the mold was taken out and the crosslinked material was recovered as a jelly tube ready to freeze-drying. The freeze-drying process was conducted as follows: a) freezing cycle consisted of a cooling phase to reach −20 °C in 5 h 30 min, that is −0.1 °C/min, followed by a stationary phase of 1 h 30 min at −20 °C, b) primary freeze-drying at 0.050 mbar (−18 °C were reached in 4 min, that is +0.5 °C/min and then temperature rose up to −5 °C in 2 h 10 min, corresponding to +0.1 °C/min rate), c) a steady state of 8 h at −5 °C, then 10 °C were reached in 1 h 15 min, that is +0.2 °C/min. At the end of freeze-drying cycle tubes were immerged in phosphate buffer saline (PBS) for 10 min under magnetic stirring and taken out from the rod to be cut. Each tube was kept in PBS at 4 °C until implantation. Tube final internal and external diameters were 1.4 mm and 1.8 mm, respectively.

For some characterization tests, materials presenting the same composition were prepared as 2D films. In that case, after adding the crosslinker, the solution was poured within two glasses separated by a silicon spacer. The final thickness of the films was 0.4 mm.

For the aging studies, 2D films and tubes were kept in PBS at 37 °C or 4 °C under static conditions.

### Scanning electron microscopy

The luminal surface of the tubes was assessed by scanning electron microscopy (SEM) using a Philips XL 30 ESEM-FEG at an accelerating voltage of 15 keV and pressure of 4 T. Samples were frozen at −20 °C and coated with gold/palladium. Four samples were tested for each tube composition.

### Gelatin release

PVA-gelatin hybrid tubes (n = 7) were placed in a glass flask containing 5 ml PBS at 37 °C for 61 days. After 7 days supernatant was collected and replaced by the same volume of PBS that was kept unchanged until the end of the study. Supernatant was kept at −80 °C before analysis. Quantification of released gelatin was performed using Indirect Competitive Enzyme Linked ImmunoSorbent Assay (ELISA) (IDBiotech- Immuno Diffusion Biotechnologies).

### Mechanical properties

#### Tensile response and elastic modulus

Two types of measurements were done to determine the elastic modulus depending on the shaping. The tensile properties of dry 2D films were measured using a uniaxial tensile testing machine (Instron 3745) equipped with a 10 N load cell and a crosshead speed of 10 mm/min under standard room conditions. Samples of 20 mm × 4 mm (length × width) were mounted onto pneumatic grips and stretched to failure. Force versus elongation data were recorded and the stress–strain plots were generated. Values of the stress and elongation at break were calculated as the average of 5 independent measurements (n = 4).

Ageing of the material, *i.e*. the evolution of the implants mechanical properties under simulated physiological conditions, was assessed. In order to simulate the *in vivo* conditions, the tensile properties of tubular constructs were characterized using a uniaxial tensile testing machine (Electroforce apparatus, Bose) equipped with a liquid chamber (NaCl 0.9% 37 °C) and 2 N load cell. Samples of 1 cm length were tested fully immersed. The tubular samples were mounted with a 6–7 mm length between clamps and attention was paid to avoid air bubbles in the lumen. The length at rest, L_0_, was defined by applying a slight preloading of 0.04 N prior to testing. For each sample, a displacement controlled sinusoidal stretching was applied for 500 cycles at 2 Hz (*in vivo* pulse rate can range from 0.7 to 3 Hz) between 0 and 60% of L_0_. A number of 500 cycles was applied as it was considered to produce stabilized cycles, for which the force at maximum stretch (60% strain) did not vary by more than 1–2% within 100 cycles. Cyclic stretching was applied in displacement-controlled mode. The strain range between 0 and 50–60% was chosen to give conservative testing conditions. Four samples per composition were measured and at least four measurements per sample were performed and video recorded. In these experiments, the tensile strain was calculated as (L − L_0_)/L_0_, where L is the instantaneous length of the tube. Nominal tensile stress was calculated by dividing the force by the initial cross-sectional area of the tubes using the formula 4 F/[π(d_o_^2^ − d_i_^2^)], where F is the tensile force, d_o_ and d_i_ the outer and inner diameters of the tube at rest, respectively. Due to the non-linearity of the tensile response, the Young’s moduli were calculated from the slope of the stress-strain curves at a strain of 20%.

#### Suture retention, burst pressure and compliance

To measure the suture-retention strength, we pulled a single throw of 6/0 Prolene suture (Ethicon) through the tube samples (n = 5) at a distance of 1 to 2 mm from the free edge, and progressively increased the weight (gram force) applied to the suture until rupture. The burst pressure was evaluated by increasing the hydrostatic pressure within the tube from a carbon dioxide tank until failure, and the burst pressure was recorded from the tank gauge (n = 6). Compliance C was defined as the inverse of Peterson’s elastic modulus and calculated on tubular samples by measuring the change in diameter while varying the pressure within the tube: C = (d120 − d80)/d80, where d80 and d120 are the diameters of the tube at the pressure of 80 and 120 mmHg, respectively.

### Platelet adhesion

Static platelet adhesion was measured *in vitro* using washed platelet suspensions. Blood was harvested from healthy volunteers into vacuum blood-collection tubes (Vacutainer® system, Beckton Dickinson) containing trisodium citrate acid-citric-dextrose. Washed platelet suspensions were prepared by centrifugation of the blood and analysed as previously reported^14^. In brief, platelets were suspended at a final concentration of 2 × 10^8^ platelets/mL. PVA and PVA-gelatin films were placed in contact with the platelets suspension at room temperature under rotation for 1 h. Adherent platelets were quantified by assessing the endogenous phosphatase activity using a pNPP buffer (0.1 M citrate, pH 5.4, 0.1% Triton X-100, 5 mm paranitrophenylphosphate). After addition of 25 μl of 1 N NaOH to stop the reaction, supernatants were harvested and absorbance was measured at 405 nm and data contrasted with a calibration curve. Bovine serum albumin (BSA)-coated and collagen-coated wells were used as negative and positive controls, respectively.

### Surgical procedure

The procedure and the animal care complied with the Principles of Laboratory Animal Care formulated by the National Society for Medical Research. The studies were carried out under authorization number 006235 of the *Ministere de l’Agriculture*, France. All animals were kept at 20–26 °C controlled temperature, under a regular 12 h light cycle. Food and water were provided ad libitum and environmental enrichment was assured. The studies were carried out under authorization number 006235 of the *Ministere de l’Agriculture*, France. Two senior surgeons and one junior surgeon performed the implantations. Each surgeon was trained to get minimal hematoma and complication, and to be able to perform with PVA tubes more than 50% of patent anastomosis at one week, controlled by Doppler Ultrasound (US). In total 54 male Wistar adult rats (12–15 weeks, mean weight 618 g) were assigned to five groups (PVA, PG0.5, PG1, PG2.5 and PG5) following a blinded method for the type of implant under study. The surgeons were not aware of the type of vascular implant, which appeared macroscopically identical. The histologist and the person in charge of the imaging were also blinded for the type of implant studied.

Vascular grafts consisted in tubular grafts of 10 mm length, 1.4 mm inner diameter and 1.8 mm external diameter, and were implanted into the abdominal aorta of adult male (Supplementary Video 1). Rats were anesthetized with sodium pentobarbital intraperitoneally administered (5 mg/100 g). In aseptic conditions, under an operating microscope and through a midline laparotomy, the infrarenal aorta was exposed and aortic branches in this segment were ligated with 9/0 monofilament Ethilon® nylon sutures. A 10 mm segment of the infrarenal aorta was transected, and replaced by the tubular grafts with end-to-end interrupted anastomosis using 10/0 Ethilon® sutures. The number of stitches used for each anastomosis ranged from 12 to 14. To avoid irreversible ischemia and paralysis, ligature of the infrarenal aorta did not exceed 70 min (the range of ischemia time was 37–67 min). The graft patency was visually confirmed after anastomosis. Neither anticoagulants nor antiplatelet were administered post-surgery. The rats were monitored daily for any signs of pain or disability until sacrifice. If irreversible damage occurred due to hypoxia this was visible within the first hours (less than 12 hours) and animals were automatically sacrificed and excluded from the study.

### *In vivo* imaging

Doppler-US non-invasive imaging were performed with a 14 MHz probe (PowerVisions 6000 with Ultrasound Image Workstation 300 A, Toshiba, Japan). Angiography was performed by injection of 1 mL of contrast agent, Hexabrix 320 (Guerbet, France), through the catheterized thoracic aorta and the blood flow was assessed using a Philips BV Endura device (Amsterdam, the Netherlands). 3D Time of Flight Magnetic Resonance Angiography (TOF-MRA) was also used to evaluate implant patency in the Radiology department of X Bichat Hospital in Paris (IRM 3 T General Electric). TOF-MRA is commonly performed in clinical practice and allows rapid and reliable small vessels patency evaluation without the use of contrast agent. Doppler-US and angiography were used in the short-term study after 1 week of implantation and TOF-MRA was used in the long-term study for imaging after 4, 8 and 12 weeks of implantation.

### Graft explantation, tissue processing and histology

Under general anaesthesia, the aorta was clamped to permit the harvesting 3 mm above and under the vascular implant, immediately after rinsing the whole with saline 0.9% without any leakage of the lumen of the tube. Microvascular implants were then fixed in 4% paraformaldehyde solution, embedded in OCT (Optimal Cutting Temperature compound) and frozen in isopentane cooled in a liquid nitrogen bath. A cryomicrotome was used to prepare 6 μm thick transverse sections of the grafts from the middle of the tube, as well as longitudinal sections. Samples were stained with hematoxylin eosin following a classic protocol: Mayer hematoxilin for 4 minutes, rinsing in tap water, differentiation in 1% acid acetic solution, rinsing in tap water, stain in eosin for 2 minutes, rinsing in tap water, dehydration and mounting. Masson’s trichrome staining for collagen fibers was conducted as follows: Mayer hematoxilin for 10 minutes, rinsing in tap water, Fuchsin-Ponceau solution for 5 minutes followed by differentiation in phosphomolybdic-phosphotungstic acid solution for 5 minutes, light green for 5 minutes, rinsing in 1% acetic acid, dehydration and mounting. Orcein to for elastic fibers observation was performed by 15 minutes in orcein solution, rinsing in tap water, rinsing in 1% lithium carbonate, dehydration and mounting. Calcium in the tissues was detected using Alizarin red staining: 2 minutes in alizarin solution, 20 seconds in acetone, dehydration and mounting. After staining, NanoZoomer digital slide scanner was used for image analysis.

Endothelial cells were visualized using RECA1 primary antibody (1:100, MCA970Serotec) and Alexa Fluor® 594 secondary antibody (1:100, A11005 Invitrogen). Smooth muscle cells were visualized using α-smooth muscle actin primary antibody (Ab5694, 2 µg/mL, AbCam) and Alexa fluor 488® secondary antibody (1:50, A11034 Molecular Probes). Nuclei were stained with DAPI (Sigma, 1:10,000). Negative control sections were incubated with the secondary or isotypes antibodies from BD Biosciences Pharmingen (Le Pont de Claix, France). Representative immunofluorescence images were taken using a Leica DMRXA microscope (Leica Microsystems).

### Statistical analysis

Data are presented as mean ± standard error of the mean (s.e.m.). One-way analysis of variance (ANOVA) with Bonferroni’s post hoc test was used to examine the significance of results between groups. The Kruskall-Wallis test was used to compare nonparametric values. A value of p < 0.05 was considered as statistically significant.

## Electronic supplementary material

Supplementary Video 1

Supplementary Video 2

Supplementary Video 3

Supplementary Video 4

Supplementary information
