# Supplementary material for: Design, characterization and in vivo performance of synthetic 2 mm-diameter vessel grafts made of PVA-gelatin blends
Source: Sci Rep. 2018 May 9;8:7417. doi: 10.1038/s41598-018-25703-2 (PMC5943294; doi:10.1038/s41598-018-25703-2)
Supplement: Supplementary file 5 — Supplementary information [file 41598_2018_25703_MOESM5_ESM.pdf]

Design, characterization and *in vivo* performance of synthetic 2mm-diameter vessel grafts made of PVA-gelatin blends

M. Atlan[1,2]†, T. Simon-Yarza[1]†, J.M. Ino[1], V. Hunsinger[1,2], L. Corté[3,4], P. Ou[1], R. Aid-Launais[1,5], M. Chaouat[1,6], D. Letourneur[1]

[1] *INSERM U1148, Laboratory for Vascular Translational Science; X. Bichat Hospital, Paris Diderot University; Paris 13 University, 75018, Paris, France*

[2] *Faculty of Medicine, University Pierre et Marie Curie; Plastic Surgery Department, Hôpital Tenon, Paris, France*

[3] *MINES ParisTech, PSL Research University, MAT - Centre des Matériaux, CNRS UMR 7633, BP 87 91003 Evry, France.France*

[4] *ESPCI-Paris, PSL Research University, Matière Molle et Chimie, CNRS UMR 7167, Paris 75005, France.*

[5] *FRIM, INSERM UMS 034 Paris Diderot University; X. Bichat Hospital, 75018, Paris France*

[6] *Plastic Surgery Department, Burn Unit, Paris Diderot University, Hôpital Saint Louis, Paris, France*

† *Equal contribution*

**Corresponding authors:*
Michael Atlan : Michael.atlan@aphp.fr
Teresa Simon-Yarza : teresasimonyarza@gmail.com
Tel: +(33)140258600
Inserm U1148, 46 rue Henri Huchard, 75018, Paris, France

Additional Information

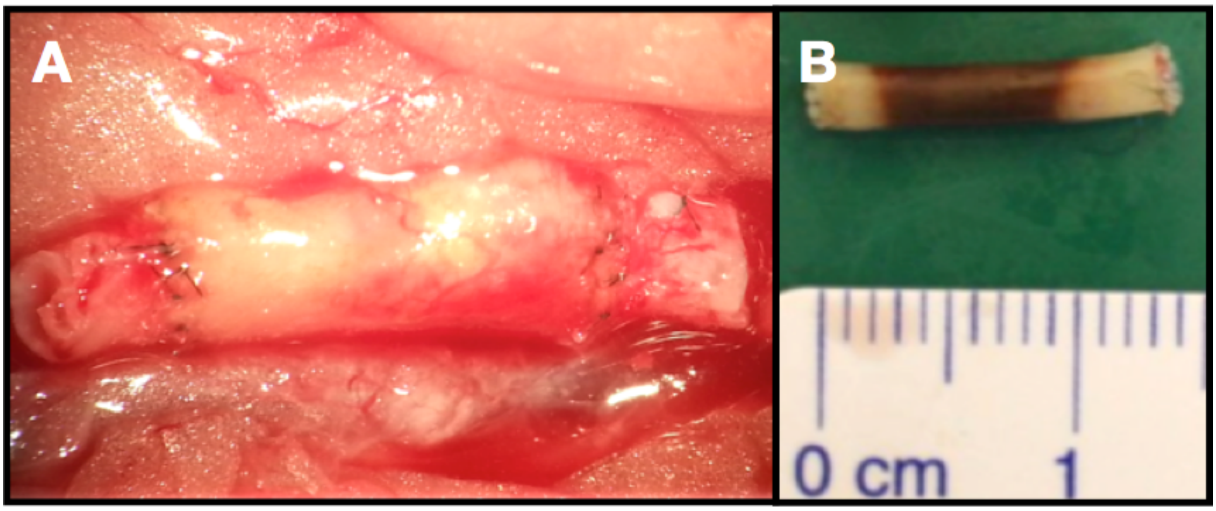

Supplementary Figure 1. A: Specimen (3mm above and below anastomosis) harvested after 4 weeks surrounded by a 200-300 µm foreign body capsule. B: Harvested grafts maintained their tubular shape, without observation of aneurysm, dilatation or any other major deformation.
